# Sustainable kidney care delivery and climate change – a call to action

**DOI:** 10.1186/s12992-022-00867-9

**Published:** 2022-08-03

**Authors:** See Cheng Yeo, Xi Yan Ooi, Tracy Suet Mun Tan

**Affiliations:** Department of Renal Medicine, Tan Tock Seng Hospital, 11 Jalan Tan Tock Seng, Singapore, 308433 Singapore

**Keywords:** Climate change, Haemodialysis, Kidney care, Sustainability

## Abstract

The delivery of kidney care, particularly haemodialysis treatment, can result in substantial environmental impact through greenhouse emissions, natural resources depletion and waste generation. However, strategies exist to mitigate this impact and improve long term environmental sustainability for the provision of haemodialysis treatment. The nephrology community has begun taking actions to improve the environmental sustainability of dialysis, but much work remains to be done by healthcare professionals, dialysis providers and professional organisations.

## Background

**Fig. 1:**
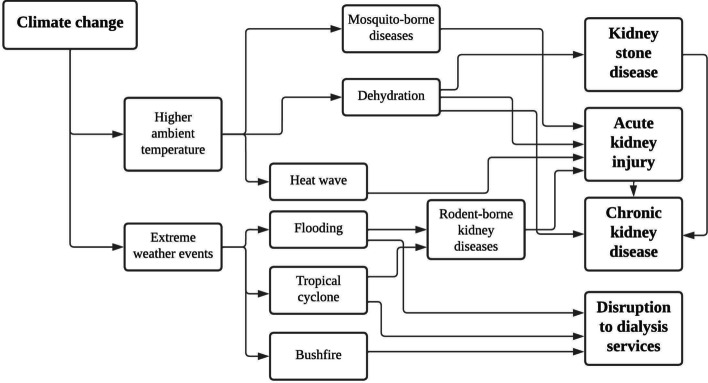
Effects of climate change on kidney health

The effects of climate change on global health are increasingly recognised [1]. Global warming, extreme weather events, and many other threats are resulting in changing patterns of disease, with direct and indirect detrimental impact on human health worldwide. Chronic kidney disease (CKD) is increasingly recognised as a global health problem, with an estimated 700 milllion people worldwide living with CKD in 2017 and its prevalence rising 29% between 1990 and 2017 [2]. People living with kidney disease are uniquely vulnerable to the effects of climate change, resulting in rising incidence of heat-related acute kidney injury and CKD, increased risk of nephrolithiasis, vector-borne kidney diseases, and disruption to kidney care delivery associated with extreme weather events (Fig. 1) [3].

Ironically, the delivery of healthcare is also a significant contributor to greenhouse emissions and natural resources depletion, where it is estimated that almost 5% of the global carbon footprint is produced by the healthcare sector [4]. Greenhouse gas emissions arise directly from healthcare facilities, as well as indirectly from the supply chain of healthcare goods and services. The relative contribution of different clinical areas of the healthcare sector to greenhouse emission is not well studied, but more recent studies have been undertaken to better understand the variability observed [5, 6]. Importantly, variation in greenhouse gas emissions per capita does not correlate highly with health system quality, suggesting that the healthcare sector’s environmental footprint can be reduced without compromising quality. While the global community adopts adaptation and mitigation strategies to minimise the impact of climate change, it is imperative that the medical community limits the carbon footprint of the healthcare sector to move towards a more sustainable healthcare delivery.

The global nephrology community has an important role to play in exploring environmentally sustainable kidney care delivery, especially since the provision of haemodialysis has been highlighted for its disproportionately high and recurrent consumption of water & electrical resources, waste generation and carbon footprint [7]. Moreover, the incidence of dialysis has increased by 43% between 1990 and 2017 [2], and more than 5 million people globally are estimated to require haemodialysis treatment by 2030, driven by rising prevalence of CKD and its risk factors such as diabetes and obesity [8]. Yet, many healthcare professionals may not realise the enormous scale of environmental impact of haemodialysis, and more importantly, the opportunities that exist for improvement [9].

### Environmental impact of haemodialysis

#### Water consumption and wastage

The provision of haemodialysis treatment requires large volumes of high-quality water to constitute the dialysate. A typical dialysate flow rate of 500 ml per minute during haemodialysis treatment necessitates 120 l of water over each typical 4-hour session. For a patient on a thrice weekly haemodialysis schedule, this equates to 18,720 l of water per patient annually. Yet, this is an under-estimation of the amount of water used to generate dialysate, as source water typically undergoes reverse osmosis (RO) filtration to remove contaminants. Current RO systems are inefficient; between 50 and 70% of the source water at the RO membrane is rejected. Thus, in a typical 4-hour dialysis session, 240 l of source water is required to prepare the dialysate. Including water required for the pre-treatment priming, rinsing and sterilisation of the system, it has been estimated that water consumption in each session of haemodialysis may be as high as 500 l [10].

#### Power

Haemodialysis systems consume large amounts of electrical power to provide equipment start-up, priming, haemodialysis treatment session, rinse and disinfection cycles, and to drive the central RO system. In Australia, the electricity consumption was estimated to be 12.0–19.6 kWh per dialysis treatment session versus 18.7 kWh per day in an average household [7]: a substantial amount considering that each dialysis machine typically provides 2 to 3 treatment sessions per day, six days a week.

#### Waste generation

Single-use dialysers, blood tubing and dialysate concentrates are often individually packed in plastic and cardboard packaging. It was estimated in one study that each haemodialysis treatment generates 2.5 kg of hazardous waste, of which 38% is plastic [11]. Another study reported up to 8 kg of waste per treatment, of which less than one-third was potentially recyclable [12].

#### Carbon footprint

Although the waste generation, water and power usage during haemodialysis is staggering, there is evidence to suggest that the largest contribution to carbon dioxide (CO_2_) emission, in fact, arises from pharmaceuticals and medical equipment necessary for haemodialysis. Studies have examined the direct and indirect effect of haemodialysis treatment on CO_2_ emission (including water, energy, consumables usage, and transportation of staff and patients). In the United Kingdom (UK), haemodialysis is estimated to result in 3.8 tCO_2_-eq emissions per patient annually [13], more than 7-fold the mean per patient carbon footprint in UK healthcare [14]. In Australia, haemodialysis treatment alone has been estimated at 10.2 tCO_2_-eq per patient annually, accounting for more than two-thirds the estimated Australian mean annual per capita CO_2_ emission of 15.4 tCO_2_-eq [11].

### Opportunities

There are several areas that can potentially mitigate the environmental impacts of haemodialysis, many of which are ready for immediate adoption, but some require further study (Table 1) [15]. One of the key areas identified is the recycling of RO reject water, since RO reject water has been shown to be as safe as source water, and recycling can be easily accomplished through redirection into storage tanks, instead of drains [16]. Significant reduction in cost of renewable power technology, such as solar rooftops, has made renewable power generation an economically viable option [17]. Retrofitting of heat exchangers to dialysis machines and upgrading to water treatment plants may reduce power and water usage respectively. Lastly, waste segregation, recycling and minimisation, through installation of baling machines to recycle plastic and cardboard, or the use of central delivery of acid for haemodialysis to reduce plastic packaging, have been undertaken.

**Table 1:** Opportunities that individual dialysis units can implement to promote sustainable kidney care

**Environmental impact of dialysis & Opportunities for improvement**	
**Water usage**	*Remarks*
Recycle reverse osmosis (RO) reject water.	Easy to implement infrastructure project with huge potential savings.
Upgrade to water treatment plants that are more efficient and allow RO reject water recirculation.	Decreases water wastage from RO reject water; significant capital investments involved.
Incremental dialysis or reduction of dialysate flow rate, where appropriate, to reduce water demand.	Dependent on patients’ clinical condition, and therefore not suitable for all patients.
**Power usage**	
Renewable energy generation eg. use of rooftop solar panels.	Requires capital investment through installation or retro-fitting equipment.
Equip dialysis machines with heat exchangers.	
**Waste generation**	
Central dialysis delivery system to reduce reliance on individually packed dialysis consumables.	Results in fixed dialysate composition with no opportunity to provide individualized care to cater to patients’ unique needs. Risks of system dysfunction which may in turn affect all patients. Risks of microbial contamination with the use of long dialysis piping.
Waste segregation for appropriate disposal & recycling.	Appropriate segregation of waste minimizes the need for incineration and/or disinfection prior to waste disposal.
Baler machines for recycling cardboard and plastic waste from dialysis consumables.	Recycling reduces the burden on landfill sites.
**Others**	
Paperless clinical documentation & laboratory reporting. Tele-health for clinicians’ review. Promotion of active modes of transport to staff and patients. Use of greener alternatives in dialysis facilities eg. motion-sensor energy-saving lights, dual-flush system in toilets, biodegradable linen etc.	Process and care model innovations, coupled with general green measures, are usually cost-free (or low cost) and can be implemented easily across different settings. These green initiatives can reduce carbon emission, save water and electricity, reduce waste, but more importantly, create awareness for healthcare professionals and patients.

The consideration of alternative dialytic therapies such as home haemodialysis or peritoneal dialysis may offer some opportunities for reducing the emissions involved with transportation of staff and patients. However, this is quickly offset by the increased frequency and duration of home haemodialysis treatments [13], and the overall plastic waste generation from peritoneal dialysis [18].

### Challenges & barriers

For many healthcare professionals around the world, the immediate priority is to deliver safe and quality care to patients, with little focus on the associated environmental impact. Even with greater awareness, healthcare professionals may be overwhelmed by information such as “CO_2_ emission per capita”, or fail to identify the relevance and/or urgency of such issues. Furthermore, the perceived additional costs required to deliver more environmentally friendly care is often a barrier to the adoption of sustainable solutions.

More broadly, people living in low- and middle-income countries (LMICs) are disproportionately susceptible to kidney disease, in part due to contributions from the social determinants of health. Besides an increased prevalence of kidney disease, people in LMICs are further at risk due to limited access to quality kidney care and kidney replacement therapy, resulting in greater health burden and worse outcome. The economic cost of climate change interventions may also be incommensurate to the limited resources faced by LMICs. Yet, people at socioeconomic disadvantage bear the greatest burden of climate change. As an example, kidney disease may be associated with occupations involving exposure to extreme temperatures, such as farming, which are disproportionately held by people in LMICs and/or lower socio-economic status.

Many of the interventions already described utilise existing technology and are easy to implement, with no compromise to delivery of basic healthcare to patients. Collective guidance from professional organisations and coordinated action from industry partners are pivotal to ensure successful implementation of sustainability policies and targeted actions [19, 20]. Moreover, there is evidence that many of the changes that are necessary to improve environmental sustainability in healthcare will also result in significant longer term cost savings and provide financial sustainability over time [21].

## Conclusion

In conclusion, healthcare professionals, dialysis providers and professional organisations should recognise the relevance and urgency of the impact of haemodialysis on climate change and can make immediate and clear contributions towards achieving more sustainable haemodialysis provision and kidney care delivery.

## Data Availability

Not applicable.

## Abbreviations

CO_2_: Carbon dioxide
CKD: Chronic kidney disease
LMICs: Low- and middle-income countries
UK: United Kingdom
RO: Reverse osmosis
